# Local knowledge as a tool for prospecting wild food plants: experiences in northeastern Brazil

**DOI:** 10.1038/s41598-020-79835-5

**Published:** 2021-01-12

**Authors:** Patrícia Muniz de Medeiros, Gabriela Maria Cota dos Santos, Déborah Monteiro Barbosa, Laílson César Andrade Gomes, Élida Monique da Costa Santos, Rafael Ricardo Vasconcelos da Silva

**Affiliations:** Laboratóry of Biocultural Ecology, Conservation and Evolution, Universidade Federal de Alagoas, Campus de Engenharias e Ciências Agrárias. BR-104, Rio Largo, AL 57100-000 Brazil

**Keywords:** Plant ecology, Biodiversity, Nutrition

## Abstract

This study aims to provide a simple framework to identify wild food plants with potential for popularization based on local knowledge and perception. To this end, we also characterized the distribution of this knowledge in the socio-ecological system. We developed the study in the rural settlement Dom Hélder Câmara in northeastern Brazil. The species with the greatest potential for popularization considering the attributes accessed from local knowledge and perception were *Psidium guineense* Sw., *Genipa americana* L., *Xanthosoma sagittifolium* (L.) Schott and *Dioscorea trifida* L.f. However, the high variation in local knowledge on wild food plants suggests that species that are not frequently cited can also be promising. The absence of age or gender-related knowledge patterns indicates that studies for prospecting wild food plants in similar socioecological contexts need to reach the population as a whole, rather than focusing on a specific group.

## Introduction

Wild food plants are a biocultural heritage of societies that know and consume them^1^, being important components of the food of several people around the world^2^. Its popularization has the potential to contribute to food diversification, which usually increases the diversity of nutrients in the diet^3^. Thus, many of these plants have significant amounts of micronutrients, which can be scarce in high-global production crops^2^. In addition to health and diet benefits, wild food plants are free from the use of pesticides and fertilizers, which is especially important in countries that maintain a significant consumption of these inputs, as is the case of Brazil^4^.

In Brazil, most wild food plants have been incorporated into the concept of “unconventional food plants” (UFP). UFP comprise plants or parts of plants that serve as food, but whose nutritional potential is unknown or underused by the majority of the population of a determined area, region or country. The term has spread to Brazilian society^5^, especially among practitioners of agroecology and healthy food consumers. In addition, in some regions of the country, plants under this category are also being used for *haute cuisine*^6,7^.

Despite the widely adoption of the term, many people have mistakenly assumed that UFP are only ruderal or short-lived plants^5^, which excludes many native and/or woody plants. This misconception may be related to the fact that most of the plants that have been popularizing under this label are leafy vegetables^5^ occurring in anthropized areas.

Therefore, a challenge for research in Brazil is to provide theoretical bases and develop mechanisms to increase the visibility of other wild/unconventional food plants, especially those that are known, consumed and commercialized, albeit in an incipient way, by communities in view of the fact that their popularization can contribute strongly to the generation of income for farmers and extractivists^7^.

To this end, efforts should be directed towards identifying plants with the greatest potential for popularization. In this sense, it is necessary that the prospecting of wild food plants for possible nutritional, economic and sensory studies are guided by information from local knowledge. This focus, although present in studies with wild food plants (e.g., Jacob et al.^8^), is much more evident in the literature on medicinal plants, which makes strong use of the ethnodirected approach to guide the prospecting of species. Therefore, it is necessary to strengthen the ethnodirected approach to prospect wild food plants without reducing this process to a mere identification of promising plants, but, alternatively connecting the plants with their biocultural heritage and strengthening the communities that hold the knowledge about them.

Thus, this study aimed to provide a simple framework to identify wild food plants with potential for popularization based on local knowledge and perception, especially among potential consumers in urban areas. Such identification can help intensifying the trade of these plants by farmers and extractivists and contribute to income generation in the field. We based our framework on identifying attributes that may influence the odds for popularization and, subsequently, we focused on how people value the species for each attribute. Previous studies included some of the attributes proposed in this study. Pieroni et al.^9^, for example, introduced a “cultural food significance index,” which considered, for example, the species’ availability, taste, and medicinal role. We used such attributes, together with several others, to find species with popularization potential.

In this study we used local knowledge and perception and, therefore, we also consider it necessary to characterize the distribution of this knowledge in the socio-ecological system.

Therefore, in order to understand aspects related to the knowledge and potential for popularizing wild food plants, this study was conducted in a rural settlement in northeastern Brazil. We departed from the following questions: (1) what is the profile of the plants known to residents in terms of habit, place of occurrence and part used?; (2) how is knowledge about wild food plants distributed?; (3) which plants have the potential for popularization among potential consumers in the surrounding urban area?; and, (4) is the local perception about the biological and ecological attributes of plants a good predictor of knowledge about these plants?

Some studies also point out that ethnobiology should improve its dialogue with other sciences (such as nutrition) to better deal with food policies and strategies^10^. Therefore, based on the available literature, we discussed the main nutritional characteristics of the top-4 species with popularization potential. We also discussed other species’ attributes that could increase (or decrease) the odds for popularization.

## Local knowledge on wild food plants

To make use of local knowledge and perception in the search for promising species, it is necessary to understand their characteristics. Local ecological knowledge is dynamic and adaptive^11^, varying in time and space. Therefore, it is common to find, to a greater or lesser degree, heterogeneity in knowledge between different locations and between individuals in the same location.

Studies have identified different patterns of distribution of local botanical knowledge and the means the routes of transmission can influence the degree of heterogeneity of this knowledge. Considering the classic models of cultural transmission, the vertical route (knowledge transmitted between different generations of the same family line) usually leads to a more conservative and heterogeneous knowledge in a socio-ecological system, that is, with less changes within the family line, but with many differences when considering the different families^12,13^. On the other hand, when horizontal (knowledge transmitted between people of the same generation) and oblique (transmission between people of different generations and different family lines) predominate, the traditional botanical body of knowledge is usually more dynamic (with greater changes over time) and less heterogeneous, considering the system as a whole^12,13^.

In addition to the routes of transmission, socioeconomic variables have been reported in the literature as determinants of traditional botanical knowledge, and age and gender are among the most important. Many studies, within the scope of food plants or plants with other uses have found that the older the person, the greater the local botanical knowledge, measured mainly from the number of known plants^14,15^. However, a growing number of studies have shown that this knowledge grows with age to the point where it stabilizes or declines, possibly due to issues associated with memory impairment in elderly people^16,17^. In addition, in the specific case of knowledge on wild food plants, there is a substantial variation reported in literature, as studies have also identified no connection between age and knowledge^18–20^. Therefore, it is important to expand the cases studied, in order to identify in which socioecological contexts this relationship gains or loses strength.

Gender is also an important predictor of local botanical knowledge due to the divisions of social roles between men and women. However, this relationship varies according to the socioecological context. A meta-analysis carried out with studies that investigate the role of the gender on the knowledge of medicinal plants showed that, while in some places (such as Brazil), women were the main holders of this type of knowledge, in others (such as in Ethiopia), men were the ones with the greatest knowledge on medicinal plants^21^. In the case of wild food plants the findings were variable. Some studies identified men as the greatest experts^15,19^, while other studies identified women as the most knowledgeable^20^ or did not find differences related to gender^18,20,22^.

Sometimes studies on local knowledge are interested in understanding the factors that make certain plants more popular than others. In this sense, the literature has indicated aspects such as taste^23–25^, availability^24^ and nutritional value^26^ as important predictors. However, these aspects have rarely been evaluated in an integrated manner and through statistical modeling.

## Methods

### Study area

**Figure 1:**
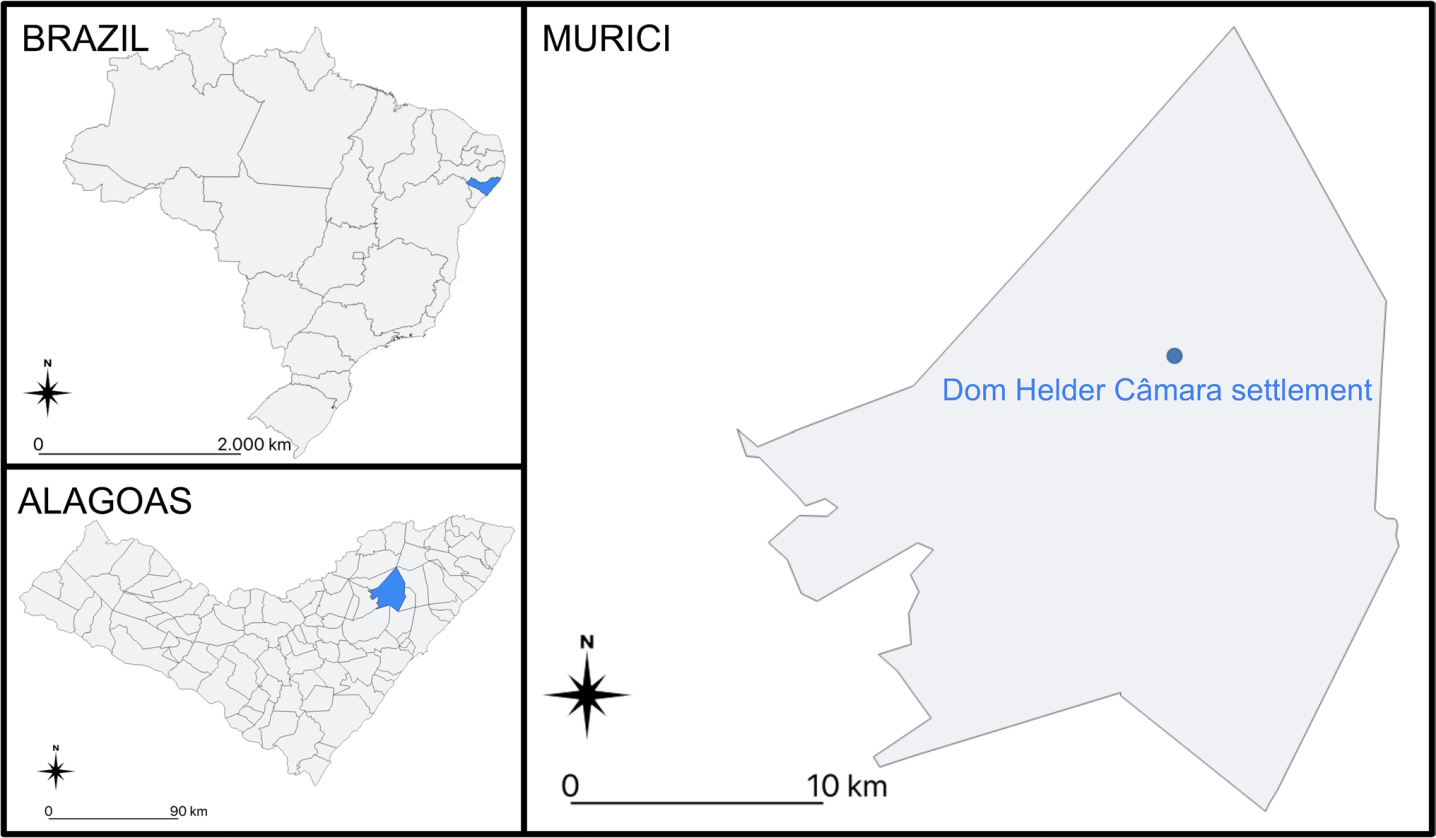
Location of the Dom Helder Câmara rural settlement, in the municipality of Murici, state of Alagoas, northeastern Brazil.

We conducted the study in the rural settlement Dom Helder Câmara, located in the municipality of Murici, State of Alagoas, northeastern Brazil (Fig. 1). The central coordinates of the settlement are 9° 15′ 18ʺ S and 35° 54′ 35″ W. Dom Helder Câmara is 30 km away from the municipality of Maceió, capital of the state of Alagoas. The National Institute of Colonization and Agrarian Reform (INCRA) created the settlement in 2000, benefiting 43 families, although 36 of them remain on the site^27^. It is officially registered as Duas Barras, a name attributed to the place before settlers occupied it^28^. Before migrating to the site, the settlers lived in other communities in the municipality of Murici and other municipalities in the state, while some residents came from distant locations outside the state of Alagoas.

Agriculture is the main activity in the settlement. Cassava, beans, yams and corn are the crops that make the greatest contribution to local food and income^27^. About a third of families carry out certified organic production and many of the other families are experiencing an agroecological transition process^29^. Families with organic certification sell their products in markets and agroecological fairs in the capital of the state of Alagoas.

The climate of the region is humid tropical, with a rainy season between April and August^30^, and the predominant vegetation is Dense Submontane Rainforest^31^. The settlement is located within the limits of the Murici Environmental Protection Area, a conservation unit for sustainable use and is 6 km away from Murici Ecological Station, an integral protection conservation unit.

### Ethical aspects

The research was approved by the Human Research Ethics Committee of the Federal University of Alagoas (CAAE 09805618.1.0000.5013). Participants signed the Free and Informed Consent Form, which authorizes the use of the information provided during the interview. All methods were carried out in accordance with relevant guidelines and regulations.

### Data collection

We made the first visits to the settlement in 2016. These visits aimed to familiarize residents with the research team and to collect preliminary information from informal conversations. Among this information, we asked some residents about what makes them consume or stop consuming a wild food plant. The responses helped to establish the attributes that will be detailed below.

We conducted individual interviews between 2016 and 2017 with 32 residents, one person per family (man or woman responsible for the residence). Four families are not represented in the study, since we did not find the heads of household at home, even after successive attempts. We initially recorded the socioeconomic data of the interviewees. The free-list technique was applied to indicate the wild food plants they know. To ensure that the interviewers and interviewees were dealing with the same cultural domain, we used the following stimulus: “what plants do you know that serve as food, are not cultivated and are present in forests, roadsides, vacant lots or inside plantations?”.

After the interviewees completed their lists, they indicated the part(s) of the plant(s) that are used as food. We used a five-point Likert scale so that participants could assign scores for each plant according to the following parameters: intensity of consumption, commercial potential, taste, nutritional potential, medicinal potential, absence of adverse effects, temporal availability of the plant’s part used as food, spatial availability of the plant’s part used as food, ease of collection and ease of propagation. To assist the interviewees, we used common terms when referring to the attributes and each point (from one to five) corresponded to a pre-established qualification (Table 1). We chose to debug the evaluations by attribute, instead of simply asking about the species with the greatest potential in general, because this detail would allow a targeted prospecting (for example, in the search for functional foods it would be relevant to obtain specific information about the perceived medicinal potential).

**Table 1:** Attributes measured by a five-point Likert scale and details of how the scale was presented to respondents from the Dom Helder Câmara rural settlement, Murici municipality, state of Alagoas, Northeast Brazil.

Atribute	Categories
Consumption intensity	1. Very little consumed 2. Little consumed 3. More or less consumed 4. Consumed 5. Very consumed
Commercial potential	1. It is not sold and cannot be sold 2. It is not sold, but it might sell 3. It is not sold, but I think it would sell well 4. It is sold 5. It is much sold
Taste	1. Very bad 2. Bad 3. Reasonable 4. Good 5. Very good
Nutritional potential	1. Feeds very badly 2. Feeds badly 3. Feeds reasonably 4. Feed well 5. Feeds very well
Medicinal potential	1. It is not medicine 2. It's bad medicine 3. It is modest medicine 4. It is good medicine 5. It is very good medicine
Absence of adverse effects	1. Always a problem when you eat 2. It is a problem frequently 3. Sometimes it's a problem 4. Almost never a problem 5. Never a problem
Temporal availability of the part used as food	1. It is found rarely in the year 2. It is found less than half of the year 3. It is found half the year 4. It is found almost the whole year 5. It is found all year round
Spatial availability of the part used as food	1. There is very little 2. There is little 3. There is a fair amount 4. There is a good amount 5. There's a lot
Ease of collection	1. It is very difficult to collect 2. It is difficult to collect 3. It's fairly easy to colect 4. It is easy to collect 5. It is very easy to collect
Ease of propagation	1. It is not possible to plant 2. It is difficult to plant 3. It is possible to plant 4. It is easy to plant 5. You don't have to do anything, it's very easy

Finally, we asked if there were plants that needed any type of treatment before being consumed and with whom the interviewees had learned about wild food plants. The plants were collected and sent to the Herbarium Dárdano de Andrade Lima of the Agronomic Institute of Pernambuco, where specialists identified them.

### Data analysis

We considered the information from *Flora do Brasil* (http://floradobrasil.jbrj.gov.br) to classify species according to their habits. The classification according to the main place of occurrence (forest × anthropic areas) was based on information from two local specialists (residents acknowledged to know the wild food plants).

We calculated the average scores for each parameter assessed during the interviews. They were calculated from the sum of the scores attributed by the interviewees divided by the number of people who mentioned the plant. Thus, to avoid bias due to idiosyncratic information, we excluded the plants that were cited by only one interviewee from the analysis. We also calculated the average overall score for each plant and the average overall score for each attribute.

Variations in knowledge about wild food plants were assessed at the individual and plant level. In the second case, we aimed to identify whether the best-known wild food plants are those best evaluated by those who know them. For this, we performed a simple linear regression, assigning the square root of the number of people who cited each plant as the response variable, and the average score of each plant as an explanatory variable, considering all the attributes. The use of a multiple regression model, considering each attribute as an independent variable, would bring more detailed results on the factors that influence knowledge. However, as the sample of known plants is small, models with high numbers of explanatory variables are at risk of presenting biases and, therefore, we chose to use the combination of all variables measured from local perception as an explanatory variable. In order to observe whether people adopt independent criteria to assign the scores or whether, alternatively, they tend to highlight the same plants, regardless of the parameter evaluated, a Spearman correlation matrix was performed between all the measured attributes.

To investigate variations in local knowledge about wild food plants, we used as parameter (1) the number of plants mentioned by the interviewees, and (2) the similarity in the lists of plants mentioned. We used the exact Wilcoxon-Mann–Whitney test to check whether there are differences between men and women in terms of the number of plants mentioned. Average rankings were used to deal with ties, due to their high incidence (equal number of plants mentioned by different people). We also investigated the relationship between age and the log_10_ of the number of plants mentioned + 1 using a simple linear regression. The transformation in the response variable served to adapt the data to the premises of linear regression.

We calculated a Jaccard similarity matrix to observe the pairwise similarity between the lists of plants mentioned. It was converted to a distance matrix to calculate a PERMANOVA, with 999 permutations, using the respondent's age and sex as independent variables.

All analyzes were performed using RStudio version 1.2.5001. We used the ‘coin’ package for the Wilcoxon-Mann–Whitney test, the ‘vegan’ package for PERMANOVA, the ‘Hmisc’ package for the correlation matrix and the ‘ggplot’ package for the correlation graph.

## Results

### Local knowledge about wild food plants

Of the 32 residents interviewed, the majority (59.4%) were women. The average age of respondents was 51.7 ± 12.4. Ages varied from 25 to 69 years. The age-class between 25 and 39 years-old represented 21.9% of the respondents, while the age-class 40–54 represented 40.6%, and the age-class 55–69 represented 37.5% of the respondents. Although all respondents were farmers, 37.5% of them started farming in the settlement 20 years ago. Most people that already performed agricultural activities before moving to the settlement worked with sugarcane cutting. Previous activities of those that were not farmers included housekeeping, homemaking and the commercialization of vegetables in local markets.

Respondents cited 26 wild food plants, of which less than half (13) were cited by more than one respondent. The most cited plants were *Dioscorea trifida* L.f. (59.4% of respondents), *Psidium guineense* Sw. (31.3%), *Syagrus cearensis* Noblick (28.1%) and *Xanthosoma sagittifolium* (L.) Schott (25%).

Of the 13 wild food plants cited by more than one respondent, the majority (69.2%) is shrub, tree or arborescent. Herbaceous plants (14.4%) and climbing plants (15.4%) were less important. Most of the plants occur in the forests of the region. However, local farmers have devoted efforts to promote these plants in gardens, backyards and other anthropic areas. Thus, there is a certain overlap in the collection sites, as 76.9% are collected in forest areas, while 53.8% are collected in the fields, roadsides, vacant lots or in other anthropic areas.

The fruit is the main part of the plants that are used as food in the region, since it was mentioned for 76.9% of the plants. The other plants have their leaves (15.4%) and tubers (7.7%) used for consumption. There was a strong specialization in consumption, in the sense that only one part of each plant was mentioned as consumed by more than one interviewee. In the case of taioba (*X. sagittifolium*), in addition to the leaf, the corm was also mentioned as food. However, it was not included in the descriptive or inferential statistical analysis because it was mentioned by only one interviewee.

Due to the high incidence of fruits, the interviewees did not mention a large number of pre-consumption treatments. Food washing was mentioned by some people, but few specific techniques could be identified and were directed only at non-fruit plants, in the sense of cooking (tubers) and scalding (leaves) before consumption.

The main route of cultural transmission of wild food plants is vertical, since 78.1% of respondents claim to have learned about them from their parents or grandparents. Learning from neighbors (18.8%) and spouses (9.4%) was substantially less mentioned.

The interviewees did not mention a high number of plants in the interviews, with an average of 3.2 ± 2.1. There were no differences between men and women regarding the number of plants mentioned (Z = − 1.3; p > 0.05). Age did not explain the number of plants mentioned (R^2^ = 0.03; p > 0.05; AIC =  − 7.23).

The average similarity between the plant repertoire cited by the interviewees was very low (0.15 ± 0.2). The similarity values varied between 0 and 1. Age (R^2^ = 0.31; p > 0.05) and sex (R^2^ = 0.29; p > 0.05) did not explain the similarity between the plants cited.

### Local perception of the potential of wild food plants

**Figure 2:**
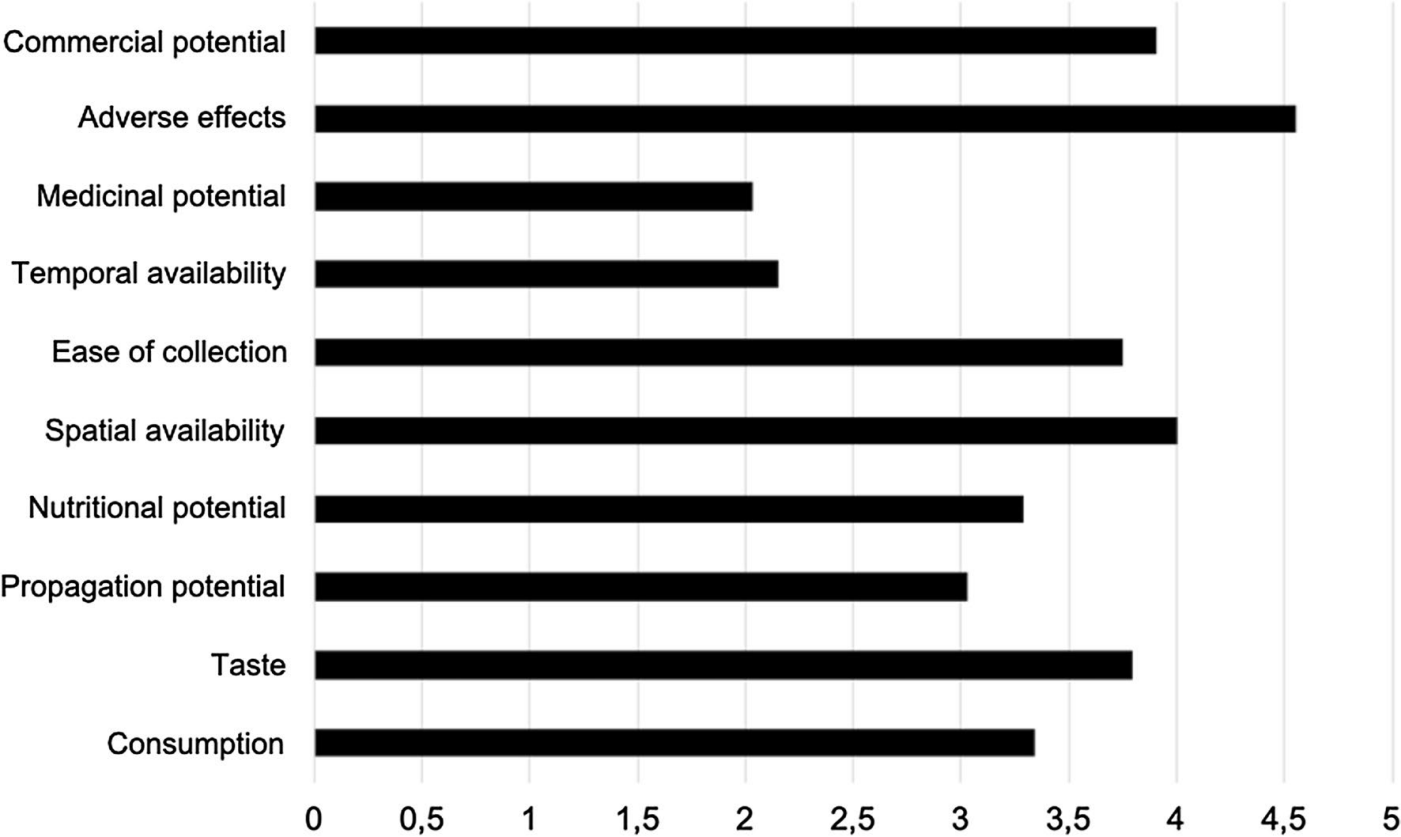
Average scores for the attributes of wild food plants according to the perception of residents of the rural settlement Dom Helder Câmara, in the municipality of Murici, state of Alagoas, northeastern Brazil.

Among all the attributes measured on the five-point Likert scale, the one with the highest overall average was the absence of adverse effects (Fig. 2), indicating that people perceive very few risks associated with the consumption of the aforementioned wild food plants. Another attribute for which the species obtained very high scores was the spatial availability. The medicinal potential and temporal availability attributes had the lowest score averages for the species (values less than 2.5). Thus, although people generally perceive wild food plants as highly available in space, they also indicate that parts of plants used as food are offered for a short period.

In general, the species average values for consumption intensity was intermediate (3.3 on a scale of one to five). The species with the highest average consumption intensity were *Couepia rufa* Ducke and *Dioscorea trifida* L.f. (Table 2). The species with the lowest average consumption intensity was *Annona coriacea* Mart.

**Table 2:** Wild food plants cited by more than one respondent in the Dom Helder Câmara community, Murici, Alagoas state, Northeast Brazil.

Species	P	Common name	Form	Env	Fr (%)	Con	Co	Tas	Pro	Nut	SA	Col	TA	Med	Ef	Ge
*Psidium guineense* Sw	Fr	Araçá	Scr/Tre	F	31.3	3.6	4.1	4.3	4.6	3.3	4.1	4.9	2.7	2.3	4.7	3.9
*Genipa americana* L	Fr	Jenipapo	Scr/Tre	A/F	18.8	3.3	4.5	3.8	3.2	3.8	4.5	3	2.3	4.3	5	3.8
*Xanthosoma sagittifolium* (L.) Schott	Le	Taioba	Her	A	25	3.1	4.1	3.4	3.8	3.9	4.4	4.5	4.8	1.4	4.1	3.8
*Dioscorea trifida* L.f	Tu	Inhame da Mata	Cli	F	59.4	4.1	4.7	4.2	3	4.5	3.2	3.8	2.1	2.6	5	3.7
*Passiflora silvestris* Vell	Fr	Maracujá	Cli	F	15.6	2.8	4.3	4.2	3.5	3	4	4	2.3	2.6	5	3.6
*Couepia rufa* Ducke	Fr	Goiti	Cli	A/F	9.4	4.7	4.3	4.3	3	3	3.7	3.3	1	3.3	5	3.6
*Amaranthus viridis* L	Le	Bredo	Her	A	15.6	3.8	4.2	4	3.4	3.5	3.2	4.8	3	1.5	3.3	3.5
*Syagrus cearensis* Noblick	Fr	Coco Catolé	Pal	F	28.1	3.7	3.4	3.8	1.5	3.3	4.7	3.5	2.5	2.4	4.6	3.3
*Annona coriacea* Mart	Fr	Aticum	Scr/Tre	A	12.5	1.8	3.8	3.3	4	4	3.3	4	1.3	1.8	5	3.2
*Syzygium cumini* (L.) Skeels	Fr	Azeitona	Tre	A/F	6.3	4	3.5	3.5	2	2	5	4.5	1.5	1	3.5	3.1
Myrtaceae (Unidentified)*	Fr	Goiabinha	Scr/Tre	A/F	6.3	3	3	4	2	3.5	5	3.5	1	1	4	3.0
*Inga* spp.	Fr	Ingá	Tre	F	21.9	2.6	3.4	3.6	2.6	2.6	3	2.9	2.1	1.1	5	2.9
*Talisia* cf. *macrophylla* (Mart.) Radlk	Fr	Pitomba da Mata	Tre	F	6.3	3	3.5	3	3	2.5	4	2	1.5	1	5	2.9

*P* part used, *Form* form of life, *Env* Preferential environment for the species to occur; *Fr (%)* frequency of citation of the species, *Con* Average score for consumption intensity, *Co* Average score for commercial potential, *Tas* average score for taste, *Pro* average score for easy propagation, *Nut* average score for nutritional potential, *SA* average score for spatial availability of the part used as food, *Col* average score for ease of collection, *TA* average score for temporal availability of the part used for food, *Med* medicinal potential, *Ef* absence of adverse effects, *Fr* fruit, *Le* leaf, *Tu* tuber, *Cli* Climbing vine, *Scr* scrub, *Tre* tree, *Her* herb, *Pal* palm, *F* forest, *A* Anthropic areas.

*Life form established from local indication and field observation.

The plants that obtained the highest averages for commercial potential were *D. trifida* and *Genipa americana* L., while the lowest average was attributed to an unidentified plant from the Myrtaceae family, known locally as *goiabinha*. Many of the wild food plants (especially the seven species with the highest score for commercial potential) are already commercialized by the interviewees in agroecological street markets in the capital. However, in most cases, plants are only offered on demand, that is, when the customer orders the product, usually in the previous week.

As for taste, *Psidium guineense* Sw. and *C. rufa* were highlighted. The species perceived as the least tasty was *Talisia* cf. *macrophylla* (Mart.) Radlk. The interviewees indicated *P. guineense* and *A. coriacea* as the most easily propagated plants, while *Syagrus cearensis* Noblick was considered the most difficult species to propagate. *D. trifida* and *A. coriacea* were regarded as the plant species with the greatest nutritional potential whereas *Syzygium cumini* (L.) Skeels was the species with the least potential.

The *goiabinha* obtained higher scores for spatial availability, while *Inga ingoides* (Rich.) Willd. was perceived as the least available. In terms of temporal availability, the most important species were those whose food use is directed to more persistent parts of the plant (*Xanthosoma sagittifolium* (L.) Schott and *Amaranthus viridis* L.). The plant with the lowest temporal availability, according to local perception, was guava.

The plants whose edible parts are easier to collect are, according to the interviewees, *P. guineenses* and *A. viridis*. The fruits of *Talisia* cf. *macrophylla* were considered difficult to collect, given the considerable height of the individuals and the persistence of the fruits on the tree. *Talisia* cf. *macrophylla* was also among the three plants with the lowest associated medicinal potential, while *G. americana* and *C. rufa* were regarded as having high medicinal potential. Seven of the 12 species obtained maximum value (5) for the absence of adverse effects. The plant that can cause more problems when consumed is, according to the interviewees, *Amaranthus viridis* L. However, if the leaves are properly scalded or boiled, their consumption does not constitute a risk.

**Figure 3:**
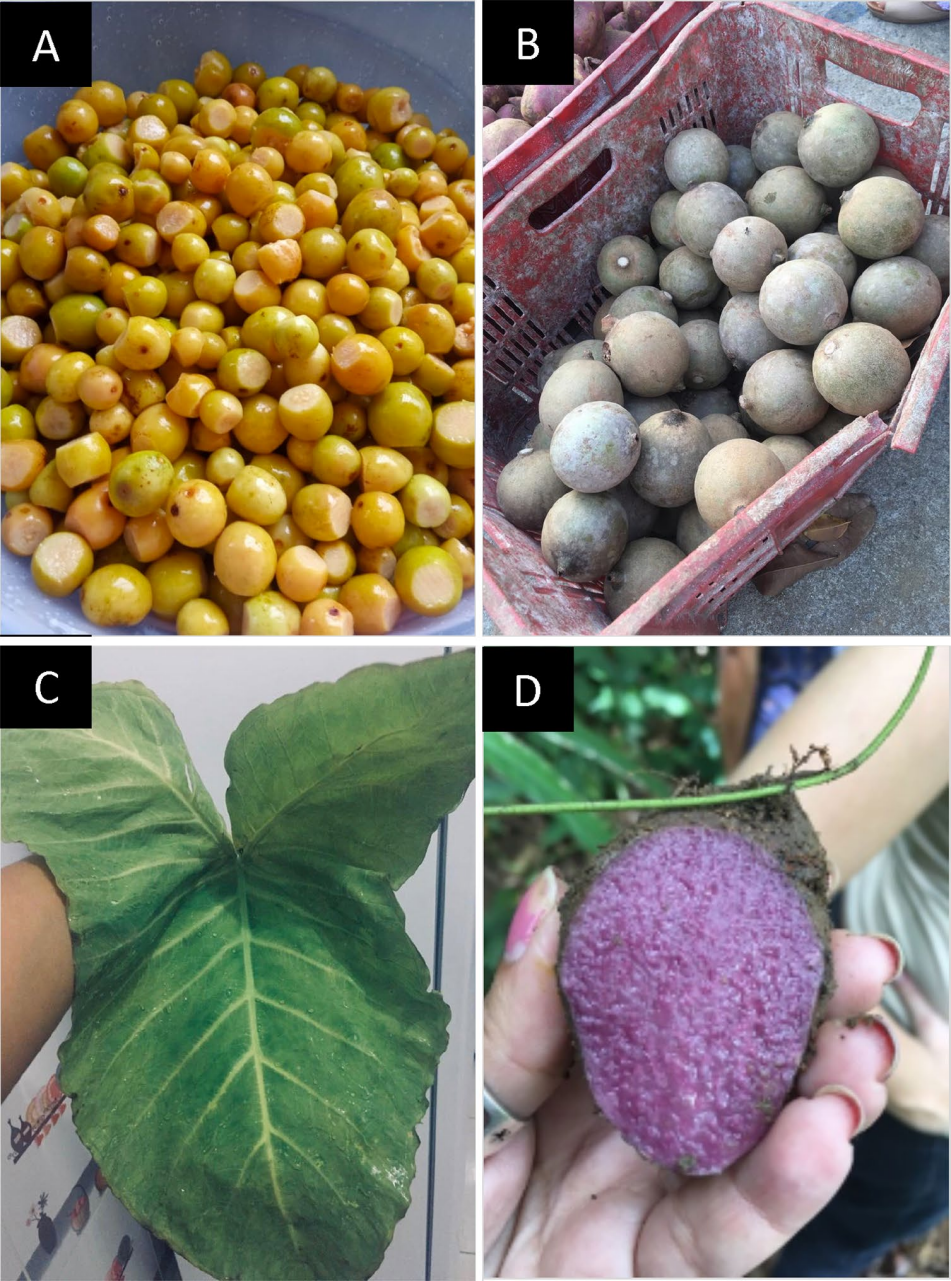
Wild food plants with greater potential for popularization, considering the average of the attributes accessed according to the perception of the residents of the rural settlement Dom Helder Câmara, in the municipality of Murici, state of Alagoas, northeastern Brazil. (**A**) *Psidium guineense* Sw.; (**B**) *American genipa* L.; (**C**) *Xanthosoma sagittifolium* (L.) Schott, and (**D**) *Dioscorea trifida* L.f.

Considering all the attributes gathered, the prominent species were *P. guineense, X. sagittifolium, G. americana* and *D. trifida* (Fig. 3).

### Perception about species attributes as a predictor of the popularity of wild food plants

**Figure 4:**
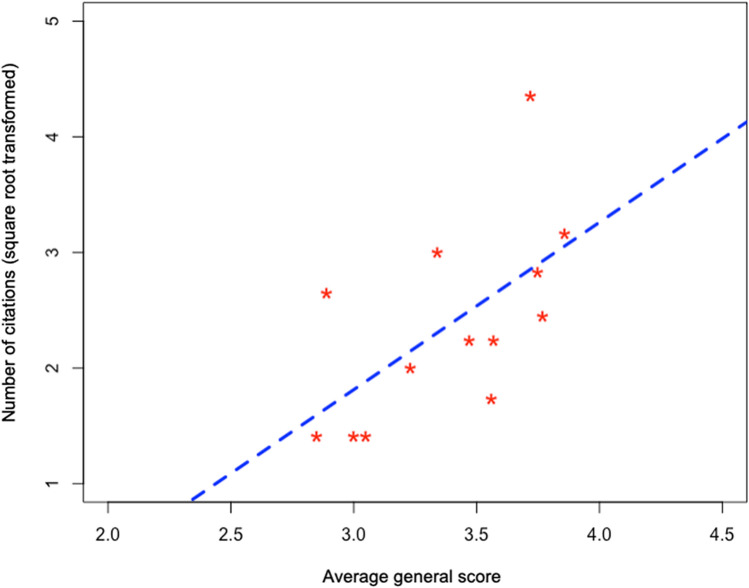
Relationship between the number of citations of wild food plants (transformed by means of square root) and the general average score of these species considering all the attributes measured according to the local perception of the residents of the rural settlement Dom Helder Câmara, in the municipality of Murici, Alagoas state, northeastern Brazil.

**Figure 5:**
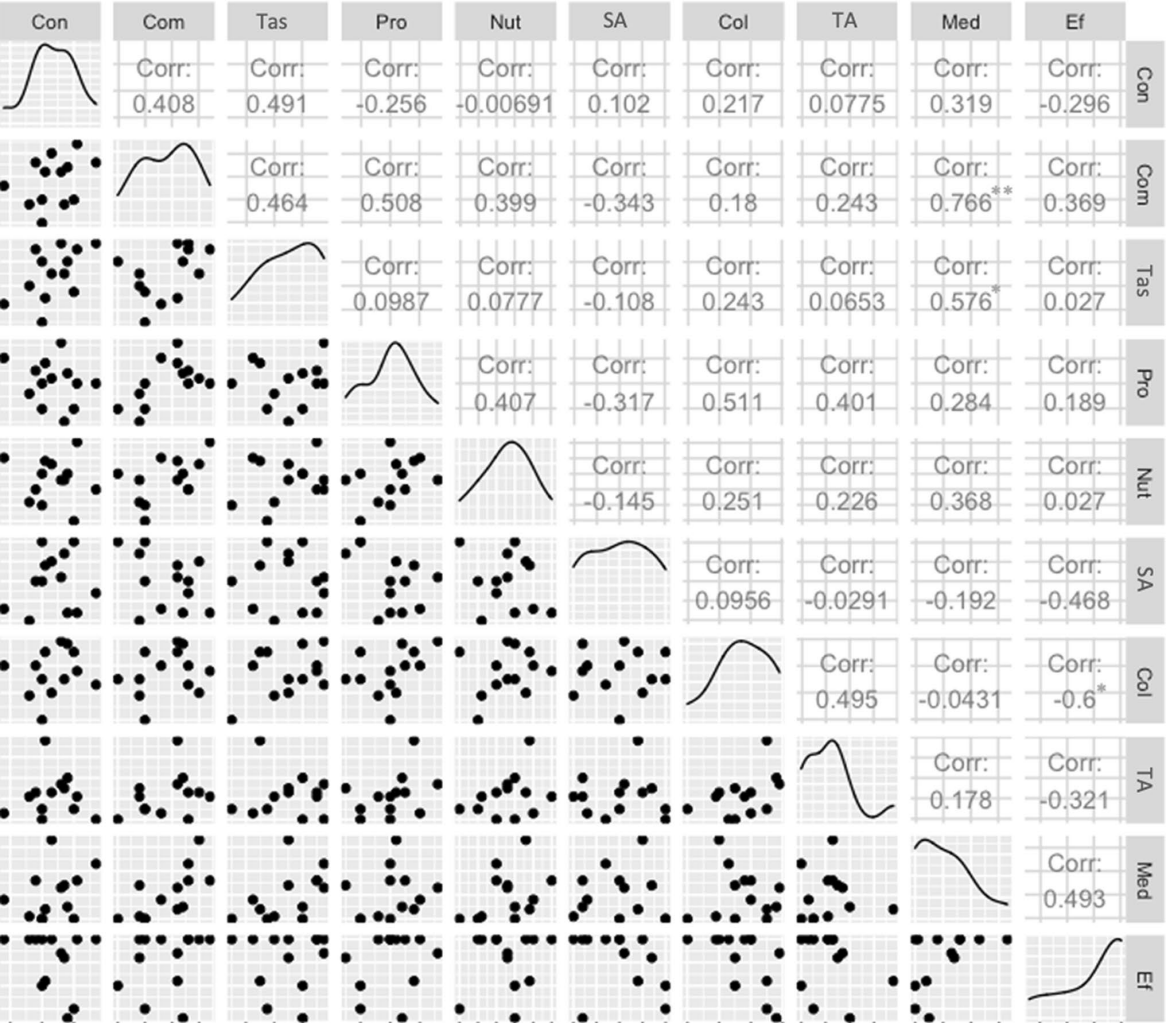
Correlogram (Spearman's correlation) between the attributes measured based on the local perception of the residents of the rural settlement Dom Helder Câmara, in the municipality of Murici, state of Alagoas, northeastern Brazil. *Cons* consumption intensity, *Com* commercial potential, *Tas* Taste, *Pro* propagation potential, *Nut* nutritional potential, *SA* spatial availability of the part of the plant used as food, *Col* Ease of collection, *TA* temporal availability of the part of the plant used as food, *Med* medicinal potential, *Ef* absence of adverse effects; *p < 0.05; **p < 0.01.

The local perception of plants' biological and ecological attributes has shown to be a good predictor of knowledge about these plants. The attributes of wild food plants influence local ecological knowledge, since the plants with the highest perceived potential are also the plants known to a greater number of people (R^2^ = 0.36; p < 0.05; AIC = 30.9) (Fig. 4). In general, the attributes measured from the local perception were not correlated (Fig. 5). This means that, when assigning scores to plants, people are actually guided by different motivations for each parameter. Exceptions were identified for the relationship between commercial potential and medicinal potential (rs = 0.77; p < 0.01), ease of collection and absence of adverse effects (rs = -0.60; p < 0.05), and taste and medicinal potential (rs = 0.58; p < 0.05). However, in none of the cases did the correlation reach values greater than 0.8, which is usually indicated as the threshold for autocorrelation.

## Discussion

### Local knowledge on wild food plants

The knowledge about wild food plants proved to be quite heterogeneous in the studied community, as the sharing of information was very low and several species were cited by a single person. These findings may be related to the nature of the community, since the community is made up of people with different geographical origins, some of whom had never worked as farmers before settling on the site.

In any case, we expected a greater sharing of information among the residents, considering that the settlement was established about 20 years ago (17 when we performed the interviews) and most of the interviewees have lived there since its origin. Thus, in addition to the diversity of previous experiences, another factor that can help explain the heterogeneity in knowledge is the predominant (vertical) transmission route. Considering that residents learned about wild food plants from their parents and grandparents (who in most cases do not live or lived there), there was no effective exchange of knowledge internally. Heterogeneity is expected when the predominant route of transmission is vertical^12,13^, however, it has certainly been intensified by the local migratory context.

When people from different geographical backgrounds converge to a place, it is plausible to expect that the richness of plants mentioned is relatively high, since each individual's biocultural knowledge would be added. However, the richness of food plants that were found in the Dom Helder settlement was lower than the values found in most studies on local or metacommunity scales in Brazil^20,32,33^ and in the rest of the world^19,26,34–36^. Thus, it is possible that during the interviews people indicated only the plants that are part of recent memory. A study on medicinal plants observed that there are temporal limits in the retrieval of information by the interviewees, so that people tend to cite mainly the plants they used in the last year^37^. Thus, memory may have served as a filter, so that, of the plants taught by parents or grandparents outside the context of the settlement, only those occurring on the site were actually mentioned.

In summary, the diversity of origins and the predominance of the vertical transmission route would have led to a strong heterogeneity in knowledge, while memory acted as a filter, in a way that only plants occurring in the settlement were mentioned. These factors together may have led to low richness and low regularity in citations. However, hypothesis-oriented studies need to address these issues.

Besides being highly heterogeneous, knowledge on wild food plants cannot be explained by socioeconomic factors. This may be partially related to the fact that people with different biocultural backgrounds converged to the community, and the dynamics of learning about food plants were different in these diverse locations, hindering the emergence of internal standards. In addition, our findings are in line with studies outside the context of settlements with regards to age^18–20^ or gender^20,32^, which indicates that, for this domain, the ways (and the moments) knowledge is acquired among individuals can vary greatly.

Regarding the profile of wild food plants, the predominance of fruits as the main part used is in line with many studies in tropical regions^20,23,33,38^, although it contrasts to most studies undertaken in temperate and subtropical regions^35,36,39–41^. This pattern can highlight the fundamental role of the environment in the selection of wild food plants. However, this hypothesis should be properly tested using meta-analytical tools.

The predominance of forest species among the wild food plants cited in the Dom Helder settlement differs from most findings in the literature for agricultural populations. According to investigations in the context of local farmers, these groups usually collect mainly species that are typical of anthropogenic areas, when compared to plants from pristine ecosystems, especially when forest areas are being lost or are distant from the community^42–44^. Studies on the role of availability in the selection of food plants have shown contrasting results^24,45,46^. In the case of the Dom Helder settlement, although anthropic areas are easier to access comparing to forests, residents have opted for a different strategy to reduce energy collection costs. Thus, instead of using primarily ruderal and invasive species, settlers chose to promote forest species in their agricultural production areas. Thus, although the forests are still the main source of collection of wild food plants, anthropic areas are gaining more and more importance, as the availability of forest species in these environments grow.

### Potential for popularization of wild food plants

The species that emerged as those with the greatest potential for popularization according to attributes of local perception have characteristics that support their choice, as indicated by the literature. However they also have attributes that can be barriers to popularization.

With the highest score for popularization potential, *Psidium guineense* is a shrub or small tree occurring in South America^47^ and Central America^48^, with wide distribution in Brazil^47^. From a nutritional point of view, the species, despite not having a high caloric content, is rich in fibers and micronutrients, especially calcium, magnesium and zinc^49^. *P. guineense* is also notiable for its content of organic acids, since among 13 species that are native to the Argentine Yungas, it obtained the highest citric acid content^50^.

Its high moisture content makes the fruit very susceptible to deterioration^49^, therefore, it requires processing (i.e. production of pulps or sweets) to reach consumers who are distant from the production sites.

In addition to having important nutritional properties, *P. guineense* also has associated medicinal properties. The ethanolic extract of its fruits demonstrated relevant antimicrobial activity against *Streptococcus mutans*, a bacterium commonly associated with cavities^51^.

*P. guineense* stood out among the interviewees of the Dom Helder settlement in terms of ease of propagation and flavor. In fact, the species is simple to propagate through seeds, with no dormancy and having optimum temperatures for germination between 20 and 25 °C^52^. With regard to taste, araçá jelly is sometimes considered superior to common guava jelly^48^. However, it is necessary to carry out sensory evaluations with different fruit products between different audiences to attest its acceptance.

*Genipa americana*, which obtained the second highest score for popularization potential, is a tree native to south and central America^53^. Similarly to araçá, it is widely distributed in Brazil^7^. The fruit has high levels of sugars, calcium and phosphorus^54^ and there are important variations in the content of macronutrients and secondary metabolites between green and ripe fruits^54,55^. The high humidity of the fruit, together with the fragility of the peel, contribute to a relatively rapid deterioration after harvest^56^, thus the processing can amplify the reach of potential consumers. There are indications that the fruit has high antioxidant activity^57,58^.

A striking feature of genipap concerns its functional properties, which is in line with the local perception that indicated the species as having the greatest associated medicinal potential. In fact, of all the plants mentioned by the residents, *G. americana* is the one that has the largest number of studies with indicative of its medicinal properties. This high medicinal versatility can be explained by the large amount of bioactive compounds in the fruits of *G. americana*^59^. Among the various compounds present in the fruit, studies have focused on the genipin and geniposide terpenes which, according to a recent review, may be useful (separetely or combined), for example, for the treatment of allergic asthma, ophthalmic diseases, viruses, cardiovascular disorders, vitiligo, depression, traumatic brain injury, Alzheimer's, diabetes, inflammation, liver ischemia and cancer^60^.

The antineoplastic activity of genipin has already been tested, for example, in cancer cells of the bladder, in order to inhibit their growth^61^. Genipin has been suggested as an efficient inhibitor of the UCP2 protein, which functions as a tumor promoter in several types of neoplasms^62^.

In addition to the medicinal potential, a blue dye can be extracted from the green fruit of *G. americana*, which has been indicated as a promising alternative to synthetic dyes, with high color and storage stability^63^. In terms of propagation, the seed has no dormancy and has more effective germination between 20 and 35 °C^64^. However, the germination process is slow, asynchronous and with low uniformity, and seedling emergence is compromised with humidity levels below 10%^65^.

The third plant in terms of perceived potential was the taioba (*Xanthosoma sagittifolium*), a herbaceous plant that is native to tropical America, but grows spontaneously and/or is grown in various parts of Africa, America, the Pacific Islands and Asia^66^. Opposing to what was observed in our study, the main part of taioba's used as food has been the corm^66^, so that the leaves are often underused. For this reason, most studies on the species are not intended for its leaves (see, for example, Akonor et al.^67^; Falade & Okafor^68,69^; Nishanthini & Mohan^70^).

Studies with the *X. sagittifolium* leaf showed high levels of protein, ash, fibers, calcium, iron and vitamin C, in addition to low energy content, which can be indicated for low-calorie diets^71^. Taioba has higher calcium levels than conventional plants such as watercress and spinach, considered important sources of this micronutrient^71^. The amount of free calcium is even higher in cooked leaves and the plant may have the potential to fight osteoporosis^72^. The boiled and lyophilized leaf is an important source of insoluble fibers and in vivo evaluations have demonstrated a reduction in the risk of colon cancer^66,73^, and protection against oxidative damage in tests performed with mice^74^.

However, the positioning of taioba as the third plant in scoring for adverse effects finds support in the literature. The species has considerable levels of calcium oxalate, a potentially toxic compound with antinutritional properties^72^. Thus, it is necessary to disseminate information to potential consumers about the forms of preparation that reduce or eliminate calcium oxalate from the leaves of taioba so that the presence of this compound does not become an obstacle to popularization. In this sense, the form of preparation of the leaf of the taioba used in the Dom Helder settlement (boiled) is precisely the most effective way to reduce calcium oxalate in conventional vegetables^75^.

The indication of taioba as one of the most easily propagated plants also finds support in the literature. Its vegetative propagation is highly efficient and can be done even with the corm division^76^. The species also has a high potential for organic production, achieving greater productivity in this system than through conventional management^77^.

*Dioscorea trifida* was the fourth species with the greatest potential according to local perception. The plant is a vine native to Central and South America^78^, is widely distributed in Brazil and has a high genetic diversity^79,80^. Although the species is not commonly grown in northeastern Brazil, it is widely grown in the Amazon region^7^.

The indication of *D. trifida* as the species with the greatest nutritional potential converges with the fact that, among the four prominent plants, it obtained the highest caloric value in nutritional studies, between 100 and 112 Kcal^7^. *D. trifida* has higher levels of iron and copper than other tubers such as yams, potatoes and macaxeira^81^. The presence of anthocyanins makes tubers a potential source of pigments for the food and cosmetic industries^78^. These pigments also have a high antioxidant activity^82^. However, considering the high genetic variability, some varieties of *D. trifida* have lower levels of anthocyanins in the tubers, and, consequently, a lower pigmentation, which seems to be the case of the strains present in the Dom Hélder settlement (inhame da mata branco and inhame da mata roxo). Therefore, it is necessary to evaluate, from a genetic and chemical point of view, the individuals of *D. trifida* occurring in the study area.

With regard to the associated medicinal importance, the species has shown potential for the treatment of food allergies^83^. In terms of propagation, its seeds have dormancy, which can be broken down effectively with storage at 28 °C for a minimum of 15 days^84^. Seed production from open and controlled pollination is easily accomplished^84^.

Because it is a species whose edible part is a tuber, its consumption can be lethal to the plant. Therefore, the incentive to cultivation must be emphasized within a program to popularize the food use for the species, in order to avoid overexploitation in areas of native vegetation.

### Perception about species attributes as a predictor of the popularity of wild food plants

Our study has accumulated evidence that knowledge about wild food plants is influenced by the biological and ecological attributes of the species. This finding is in line with other investigations that aimed to understand the factors that interfere with the knowledge and/or use of these plants^23–25,85^. In our case, we did not specifically identify the attributes of greatest interference on the knowledge about these plants. However, our study reinforces the non-randomness of knowledge and the importance of intrinsic attributes of the species in the structure of socio-ecological systems.

A possible bias in our framework could exist if the attributes measured from the local perception were highly correlated. This could indicate that people would always be assigning the highest scores to the same plants, regardless of the attribute considered, that is, without there being any greater reflections about each attribute itself. Thus, the absence of high correlations between the attributes indicated independence between the factors analyzed, which, in our view, reinforces the framework used. However, depending on the local context, highly correlated variables can be excluded and other variables can be included to access the potential of wild food plants, such as post-harvest resistance and the temporal availability of the part of the plant used for food^24^.

## Conclusion

Based on the attributes measured from local knowledge and perception, we reccomend the species *Psidium guineense, Genipa americana, Xanthosoma sagittifolium* and *Dioscorea trifida* for popularization efforts in northeastern Brazil. In this sense, it is necessary to expand studies related to the economic valuation and acceptability of products produced from these plants. However, the high variation in local knowledge about wild food plants indicates that species that are not often mentioned can also be promising. Thus, these species should also be studied from an ethnographic point of view in other socioecological contexts with less intracultural variation in knowledge.

The absence of age or gender-related patterns of knowledge indicates that there are no well-established profiles in the role of safeguarding knowledge about wild food plants. Therefore, studies to prospect these plants in places with similar contexts need to cover people with different socioeconomic profiles, instead of focusing on local specialists.

Future studies should be carried out on broader time scales, in order to cover a greater number of species and allow details on the factors that interfere on the knowledge people have about them.

We also recommend the top-4 species (and other species with potential) to be studied in terms of the social and environmental sustainability of an eventual popularization. We believe that this type of information would help local people choose which species are worth trying to popularize. We particularly advise studying (1) the ecological implications of an increase in demand, and (2) supply chain development potentialities.
